# Saponins in Chinese Herbal Medicine Exerts Protection in Myocardial Ischemia–Reperfusion Injury: Possible Mechanism and Target Analysis

**DOI:** 10.3389/fphar.2020.570867

**Published:** 2021-01-14

**Authors:** Ruiying Wang, Min Wang, Jiahui Zhou, Daoshun Wu, Jingxue Ye, Guibo Sun, Xiaobo Sun

**Affiliations:** ^1^ Institute of Medicinal Plant Development, Chinese Academy of Medical Sciences and Peking Union Medical College, Beijing, China; ^2^ Beijing Key Laboratory of Innovative Drug Discovery of Traditional Chinese Medicine (Natural Medicine) and Translational Medicine, Institute of Medicinal Plant Development, Peking Union Medical College and Chinese Academy of Medical Sciences, Beijing, China; ^3^ Key Laboratory of Bioactive Substances and Resources Utilization of Chinese Herbal Medicine, Ministry of Education, Institute of Medicinal Plant Development, Chinese Academy of Medical Sciences and Peking Union Medical College, Beijing, China; ^4^ Key Laboratory of Efficacy Evaluation of Chinese Medicine Against Glycolipid Metabolic Disorders, State Administration of Traditional Chinese Medicine, Institute of Medicinal Plant Development, Peking Union Medical College and Chinese Academy of Medical Sciences, Beijing, China; ^5^ Key Laboratory of New Drug Discovery Based on Classic Chinese Medicine Prescription, Chinese Academy of Medical Sciences, Beijing, China

**Keywords:** saponins, traditional Chinese medicine, myocardial ischemia-reperfusion injury, mechanism, ginsenosides, aralia saponins

## Abstract

Myocardial ischemia is a high-risk disease among middle-aged and senior individuals. After thrombolytic therapy, heart tissue can potentially suffer further damage, which is called myocardial ischemia-reperfusion injury (MIRI). At present, the treatment methods and drugs for MIRI are scarce and cannot meet the current clinical needs. The mechanism of MIRI involves the interaction of multiple factors, and the current research hotspots mainly include oxidative stress, inflammation, calcium overload, energy metabolism disorders, pyroptosis, and ferroptosis. Traditional Chinese medicine (TCM) has multiple targets and few toxic side effects; clinical preparations containing *Panax ginseng* C. A. Mey., *Panax notoginseng* (Burk.) F. H. Chen, *Aralia chinensis* L., cardioprotection, and other Chinese herbal medicines have been used to treat patients with coronary heart disease, angina pectoris, and other cardiovascular diseases. Studies have shown that saponins are the main active substances in TCMs containing *Panax ginseng* C. A. Mey., *Panax notoginseng* (Burk.) F. H. Chen, *Aralia chinensis* L., and *Radix astragali*. In the present review, we sorted the saponin components with anti-MIRI effects and their regulatory mechanisms. Each saponin can play a cardioprotective role via multiple mechanisms, and the signaling pathways involved in different saponins are not the same. We found that more active saponins in *Panax ginseng* C. A. Mey. are mainly dammar-type structures and have a strong regulatory effect on energy metabolism. The highly active saponin components of *Aralia chinensis* L. are oleanolic acid structures, which have significant regulatory effects on calcium homeostasis. Therefore, saponins in Chinese herbal medicine provide a broad application prospect for the development of highly effective and low-toxicity anti-MIRI drugs.

## Introduction

To date, revascularization, such as thrombolysis, is an effective method for the treatment of ischemic cardiomyopathy in patients with acute myocardial infarction (Yang et al., 2018). However, reperfusion can still cause other damage to the myocardium, which greatly reduces the advantages of reperfusion therapy (Bell and Yellon, 2011). Therefore, myocardial ischemia-reperfusion injury (MIRI) is a current clinical problem that needs urgent attention. MIRI involves a variety of classical mechanisms, including oxidative stress, inflammation, calcium overload, and mitochondrial damage (Turer and Hill, 2010). In recent years, there has been an increasing number of studies on cell pyroptosis, ferroptosis, and autophagy during MIRI (Jia et al., 2019; Li C. Y. et al., 2020). Over the past three decades, methods to reduce MIRI have been in development and have been used in clinical treatments (Garcia-Dorado et al., 2014). The treatment methods mainly include non-pharmacological interventions (ischemic pre-conditioning) and pharmaceutical treatments (Ibanez et al., 2015). At present, several drugs are effective in MIRI animal models, but their clinical use is not ideal, which may be due to the complex pathological mechanism of MIRI.

Traditional Chinese medicines (TCM) has a holistic treatment concept, and has the advantages of multiple targets, multiple links, and multiple approaches. In the field of TCM, the etiology of MIRI involves deficiency of qi, blood stasis, and phlegm. The clinical treatment is often based on TCM and compound preparations with the effects of replenishing qi and nourishing yin, warming the heart, promoting blood circulation and removing blood stasis, and expelling phlegm (Liu et al., 2013). TCM containing a large amount of saponins include *Panax ginseng* C. A. Mey., *Panax notoginseng* (Burk.) F.H. Chen, *Panax quinquefolium* L., *Aralia chinensis* L., and *Radix astragali* (Tu et al., 2013; Aravinthan et al., 2015; Wang H. W. et al., 2018). In recent years, many studies have shown that the saponins extracted from TCM has great anti-MIRI effects *in vivo* and *in vitro*; their mechanisms are diverse and mainly involve regulating energy metabolism and calcium homeostasis, and inhibiting oxidative stress and inflammation (Zhu et al., 2017; Wang et al., 2019). Saponins mainly include four-ring triterpene saponins and five-ring triterpene saponins. Among the tetracyclic triterpene type saponins, dammarane-type saponins have been studied in-depth, while among the pentacyclic triterpene-type saponins oleanane-type saponins are most widely distributed and studied (Leo et al., 2007; Hu et al., 2010; Shin et al., 2015; Zebiri et al., 2016). Each saponin also has its unique protection mechanism for MIRI due to its structural specificity.

**FIGURE 1:**
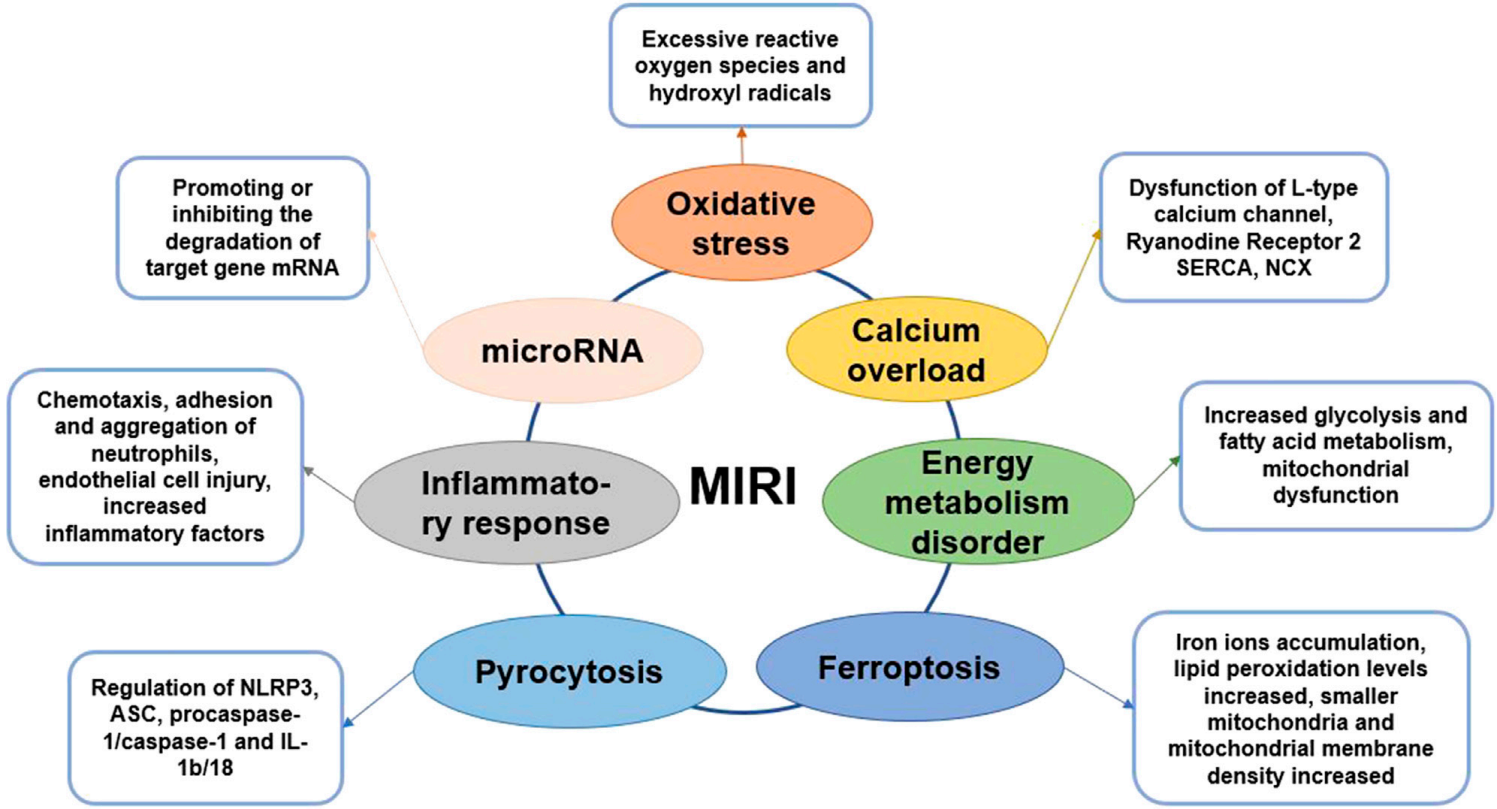
Graphical summary of MIRI mechanism.

In this review, we discuss the classic mechanisms of MIRI and a few emerging regulatory mechanisms (Figure 1). Based on the significant anti-MIRI effect of saponins, we classified and summarized the saponins with cardioprotective effects and analyzed their cardioprotective mechanisms. This review aims to provide potential treatment strategies and drug candidates for MIRI.

## Mechanisms of Myocardial Ischemia–Reperfusion Injury

### Oxidative Stress and Myocardial Ischemia–Reperfusion Injury

MIRI is accompanied by an excess of oxygen free radicals, and reactive oxygen species (ROS) are the main driving forces for reperfusion injury (Zhou et al., 2015). ROS and cellular redox states regulate many critical cellular activities. In presence of sufficient oxygen supply, ROS and endogenous antioxidants maintain balance to protect essential activities in the cell (Cadenas, 2018). MIRI damages the cellular antioxidant system and promotes oxidative damage. After reperfusion, the generation of excessive ROS, notably hydroxyl radicals, may cause the oxidation of proteins, lipids and nucleic acids (Dongo et al., 2011). This further leads to changes in protein function, membrane damage, gene mutations, and metabolic disorders, which generates oxidative stress. Superoxide anion (O^2−^), hydroxyl radical (OH^−^), and hydrogen peroxide (H_2_O_2_), which are the culprits responsible for inducing oxidative stress in the vascular wall, are mainly produced through xanthine oxidase, NADPH oxidase, endothelial nitric oxide synthase, and other enzyme systems (Zhou et al., 2015). In other words, a close connection exists between endothelial cells and ROS injury. The enhancement of endogenous antioxidant activity and the intervention of exogenous antioxidants can effectively inhibit oxidative stress and reduce damage to cells (Matsushima et al., 2014; Zhao D. et al., 2017). Therefore, researchers have screened several natural compounds with antioxidant activity, such as araloside C, dioscin, etlatoside C, ginsenoside Rb3, and ginsenoside Rg3 (Xia et al., 2011; Wang M. et al., 2015), which are expected to protect the heart by inhibiting oxidative stress.

### Inflammatory Response and Myocardial Ischemia–Reperfusion Injury

The inflammatory response activates during myocardial ischemia and is significantly aggravated during reperfusion (Vinten-Johansen et al., 2007). The adhesion and infiltration of neutrophils are the main pathological changes of coronary arteries after MIRI. After MIRI, the metabolism of arachidonic acid on the myocardial cell membrane increases, which leading the production of large amounts of leukotrienes, prostaglandins, and thromboxane A2 (Boag et al., 2017). And then the expression of special adhesion molecules on the surface of microvascular endothelial cells or leukocytes increases, which promotes the chemotaxis, adhesion and aggregation of neutrophils, increases the blood flow resistance of microvascular, and even causes no-reflow phenomenon, aggravating myocardial ischemic damage (Arslan et al., 2008). Neutrophils adhere to the endothelial cells of the blood vessel wall under the guidance of adhesion molecules and then migrate to the myocardial tissue (Moos and Funk, 2008). During MIRI, the levels of inflammatory factors, including tumor necrosis factor-α (TNF-α) and IL-1, significantly increase in the myocardial tissue causing myocardial damage. Anti-inflammatory factors and pro-inflammatory factors co-exist during MIRI, and generally, the pro-inflammatory factors are more dominant (Singhal et al., 2010).

Endothelial cells are closely related to inflammation, and endothelial dysfunction and microcirculation injury are important bases of MIRI. Studies have found that endothelial cells are more vulnerable to damage than cardiomyocytes during reperfusion, whereas cardiomyocytes are more vulnerable to damage during the ischemic phase (Singhal et al., 2010; Schanze et al., 2019). Therefore, coronary endothelial cells are critical mediators of myocardial dysfunction post MIRI. Autophagy of endothelial cells would cause structural and functional damage that hinders the blood flow to increase MIRI (Raivio et al., 2009; Russo et al., 2017). The mechanism of endothelial cells involved in MIRI may be related to the AMPK/mTOR and nuclear factor κB (NF-κB)-p65-Beclin1 pathways (Moos and Funk, 2008; Singhal et al., 2010). The role of NOD-like receptors (NLRs) with a pyrin domain 3 (NLRP3) inflammasome in MIRI is a current hot topic. NLRP3 can be combined with caspase-1 and ASC to form the NLRP3 inflammasome, which requires NF-κB activation (Wang Y. et al., 2015; Li et al., 2017). Recent research on the role of NOD-like receptor (NLR) with a pyrin domain 3 (NLRP3) inflammasome in MIRI is a hot topic. NLRP3 can be combined with caspase-1 and ASC to form the NLRP3 inflammasome, which requires NF-κB activation (Sandanger et al., 2016). NLRP3 inflammatory bodies promote the increase in IL-1β levels, triggering downstream inflammatory responses, including leukocyte recruitment and activation. Studies have shown that during the MIRI process, the activation of NLRP3 inflammatory bodies has cardioprotective effects (Vinten-Johansen et al., 2007; Sandanger et al., 2016). Some natural saponins, such as celastrol, ginsenoside Rb1, and ginsenoside Rb3, can reduce MIRI by inhibiting inflammation and restoring endothelial cell function (Wang Y. et al., 2015; Li et al., 2017).

### Calcium Overload and Myocardial Ischemia–Reperfusion Injury

As the second messenger in the cell, Ca^2+^ can maintain cardiomyocyte function, proliferation, division, energy metabolism and other important processes (Verkhratsky and Parpura, 2014). During myocardial ischemia, ATP production decreases, resulting in decreased sarco-endoplasmic reticulum Ca^2+^ ATPase (SERCA) activity (Zhu et al., 2017). Thus, intracellular calcium transport is impaired, causing calcium overload. On reperfusion, ROS damage the cell membrane, leading to extracellular Ca^2+^ influx; NCX transports Ca^2+^ into the cells and discharges a large amount of Na^+^ in the cell, exacerbating calcium overload (Ohtsuka et al., 2004). Intracellular Ca^2+^ regulates the contractile and diastolic function of cardiomyocytes and plays an essential role in excitation-contraction coupling (MacLennan and Kranias, 2003). The action potential triggers a small amount of Ca^2+^ to enter the cell through L-type calcium channels (LTCC), and a large amount of Ca^2+^ is released from the sarcoplasmic reticulum (SR) through ryanodine receptor 2 (RyR2). Then, calcium and troponin C combine to cause myocardial cell contraction (Grueter et al., 2007). SERCA can retake the intracellular Ca^2+^ to the SR, while NCX in the cell membrane can export Ca^2+^ from the cells, leading to relaxation of the cardiomyocytes (Fang et al., 2016). Therefore, the intracellular calcium level affects the excitability degree and relaxation rate of cardiomyocytes. The occurrence of calcium overload is bound to have a non-negligible effect on the contraction and relaxation of cardiomyocytes (Li et al., 2013). Calcium overload is closely related to oxidative stress, and inflammatory response and these factors together promote MIRI process (Verkhratsky and Parpura, 2014). Therefore, inhibiting calcium overload is an effective method to reduce heart damage during MIRI. Some natural ingredients, such as ginsenoside Re, ginsenoside Rb1, Elatoside C and Araloside C, can promote the restoration of calcium homeostasis and thus play a cardioprotective role (Wang et al., 2014; Wang M. et al., 2015).

### Energy Metabolism Disorder and Myocardial Ischemia–Reperfusion Injury

In the early stage of myocardial ischemia, because the oxygenated hemoglobin in the ischemic tissue is consumed, the energy metabolism changes from aerobic oxidation of mitochondria to glycolysis (Cui et al., 2017; Alegre et al., 2020). So, ATP produced by glycolysis becomes dominating source of energy to the maintenance of myocardial cell survival. The enhancement of glycolysis induces the intracellular lactate increase, intracellular pH drops, which leading the calcium overload process is aggravated. In addition, the level of fatty acid oxidation and metabolism after MIRI significantly exceeds the level before ischemia, which slows the recovery of heart function (Li et al., 2018; Tian et al., 2019). The main reason is that fatty acid β oxidation accelerates and ATP production increases, but oxygen consumption also increases (Lesnefsky et al., 2017).

To improve myocardial metabolism during MIRI, it is mainly to promote glucose metabolism and inhibit fatty acid metabolism. Optimizing energy metabolism is only a cytoprotective measure, and the therapeutic effect is limited. The treatment purpose of improving energy metabolism is to prolong the process of ischemic myocardial necrosis, buy precious time for myocardial revascularization therapy, and promote the recovery of heart function (Paradies et al., 2018). Because metabolic drugs have no obvious hemodynamic effects, it is recommended to use them in combination with β-receptor blockers. At present, there are many studies on trimetazidine and niacin, but the research progress is relatively slow due to some side effects (Wu et al., 2018). The saponins in natural products are safer to be used as medicines, and have the advantage of multiple targets. It is worth carefully exploring the effective ingredients of anti-MIRI. Studies have shown that ginsenosides including total ginsenosides, ginsenoside Rg1, ginsenoside Rb1 have a significant effect on energy metabolism (Cui et al., 2017; Li et al., 2018).

Mitochondria are the main organelles that produce ATP. Cardiomyocytes need to consume large amounts of ATP to maintain normal function. Oxygen is required for mitochondrial energy production (Alam et al., 2015). But during MIRI, the levels of oxygen reduce but those of ROS increase, which result in reduced ATP synthesis and mitochondrial permeability transition pores (MPTP) opening (Paradies et al., 2018). Therefore, MIRI causes energy metabolism disorders, and MPTP opening further promotes cardiomyocyte apoptosis and necrosis (Ong et al., 2015). In the meantime, pathways, including glycogen synthesis kinase 3β (GSK-3β) pathway, protein kinase C (PKC) pathway, signal transducer and activator of transcription 3 (STAT3) pathway and apoptosis signaling pathway, are activated successively (Makhdoumi et al., 2016; Lesnefsky et al., 2017). These pathways further affect the function of mitochondria, either promoting or inhibiting cardiomyocyte apoptosis (Zheng et al., 2017). Saponins, such as Araloside C, astragaloside IV, dioscin, ginsenoside Rb1, ginsenoside Rd, ginsenoside Rg1 and notoginsenoside R1, can reduce MIRI by regulating mitochondrial function (Wang et al., 2012; Li et al., 2018).

Mitochondrial autophagy is a process that selectively removes damaged mitochondria to reduce cell damage. During MIRI, PTEN induces mitochondrial autophagy through pathways such as putative kinase 1 (PINK1)/Parkin, BNIP3/NIX, and FUNDC1 signaling pathway (Ney, 2015). Moderate mitochondrial autophagy has a protective effect on the maintenance of mitochondrial membrane potential and the normal structure and function of cell membranes, thereby reducing MIRI. In contrast, mitochondrial dysfunction leads to impaired autophagy function, insufficient clearance, or excessive activation of mitochondrial autophagy, which can increase MIRI (Shirakabe et al., 2016; Tong and Sadoshima, 2016). Exploring mitochondrial autophagy and its regulatory mechanism during MIRI may help to understand the relationship between mitochondrial autophagy and MIRI, and provide new ideas for the clinical treatment of MIRI.

### microRNA and Myocardial Ischemia–Reperfusion Injury

In recent years, the application of microRNA (miRNA) and long noncoding RNAs (lncRNA) in MIRI treatment has increasingly become a research focus. miRNA is an endogenous, single-stranded, non-coding, small regulatory RNA in a variety of eukaryotic cells (Diaz et al., 2017). Studies have shown that miR-1275, miR-133, miR-148a and miR-324 interfere with the process of MIRI by inhibiting myocardial cell apoptosis, reducing myocardial inflammation, and promoting angiogenesis (Dai et al., 2020; Jiang et al., 2020; Zong and Wang, 2020). lncRNA is a heterogeneous non-coding RNA that can directly regulate the transcription of target genes and the degradation of proteins (Ruan et al., 2019). lncRNA has the function of competing or cooperating with endogenous RNA, which can promote or inhibit the degradation of target gene mRNA by miRNA, thereby regulating the expression of target gene mRNA and its protein (Xiong et al., 2019). The main functions of lncRNA include regulating gene methylation, transcription activation, and binding to mRNA and miRNA to affect the translation process (Pei et al., 2020). At present, most researches focus on the mechanism of lncRNA regulating miRNA. But miRNA can also regulate lncRNA. lncRNA and miRNA mainly regulate and treat MIRI through mechanisms such as oxidative stress, inflammatory infiltration, mitochondrial dysfunction, autophagy, and apoptosis (Ruan et al., 2019; Pei et al., 2020). To clarify the complex and delicate regulatory network of lncRNA-miRNA-mRNA is important for revealing the interaction between RNA molecules (Xiong et al., 2019). The role and interpretation of the complex molecular network regulation between its functions is of vital importance, providing new therapeutic targets for the treatment of MIRI.

### Pyrocytosis and Myocardial Ischemia–Reperfusion Injury

Pyroptosis is a new form of programmed cell death that is accompanied by inflammatory reactions (Bergsbaken et al., 2009). It is characterized by morphological necrosis and apoptosis. However, its features are entirely different, for example, nuclear shrinkage, DNA breakage, and a large number of 1–2 nm-diameter holes on the cell membrane are observed (Jia et al., 2019). Pyroptosis mainly depends on caspase-1 and is accompanied by an inflammatory cascade. During MIRI, ASC combines with pro-caspase-1 to form a multi-protein complex that activates caspase-1, which in turn induces the activation of IL-1β and IL-18, recruits more inflammatory factors, and expands the inflammatory response (Toldo et al., 2018; Wang Z. et al., 2018). NLRP3 is also an important factor that initiates cell pyroptosis and mediates the production of IL-1β and IL-18. In the early stage of MIRI, NLRP3 inflammation is activated before apoptosis, indicating its participation in the pathological process of MIRI, and therefore it may be considered a marker of early MIRI (Allam et al., 2014; Qiu et al., 2017; Yumnamcha et al., 2019). Hence, understanding the specific role of NLRP3, ASC, procaspase-1/caspase-1, IL-1b/18, and other proteins related to the pyroptosis signaling pathway in MIRI is essential to develop targeted cell pyroptosis and provide new ideas for MIRI prevention in the future (Yang et al., 2018; Wu et al., 2019).

### Ferroptosis and Myocardial Ischemia–Reperfusion Injury

Ferroptosis is a new form of cell death caused by MIRI (Baba et al., 2018). It is characterized by the generation of ROS through the reaction of ferritin, which causes the accumulation of lipid peroxides and manifests in iron ion accumulation, increased lipid peroxidation levels, smaller mitochondria, and increased mitochondrial membrane density (Xie et al., 2016; Fang et al., 2019). Due to its iron-dependent characteristics, ferroptosis is genetically and biochemically different from other forms of cell death. Inhibitors of apoptosis, pyroptosis, and autophagy cannot prevent the occurrence of ferroptosis, but iron-chelating agents can inhibit cell ferroptosis (Baba et al., 2018; Yumnamcha et al., 2019). Ferroptosis-induced ERS leads to apoptosis, which is closely related to MIRI; ferroptosis induces an unfolded protein response during ERS and subsequently activates the PERK-eIF2α-ATF4-CHOP signaling pathway, leading to apoptosis, which plays a vital role in the process of MIRI (Peng et al., 2020). Moreover, p53 upregulates apoptosis regulators and participates in the synergy between ferroptosis and apoptosis (Chen et al., 2019; Sumneang et al., 2020). However, although ferroptosis is closely related to MIRI, its precise molecular mechanism and biological function are not yet fully elucidated. Research on its mechanism is expected to provide new insights for MIRI treatment.

### Interaction Among Myocardial Ischemia–Reperfusion Injury Mechanisms

Due to ischemia and hypoxia of myocardial tissue, energy metabolism disorder is the initiation of MIRI (Lesnefsky et al., 2017). In addition, the ischemic and hypoxic environment creates a certain material basis for the formation of oxygen free radicals (Chen and Chow, 2005). With the massive formation of oxygen free radicals, it directly or indirectly leads to calcium overload in myocardial cells. Calcium overload can cause damage to mitochondrial structure and function under the joint participation of inflammatory (Verkhratsky and Parpura, 2014). Calcium overload and mitochondrial dysfunction are mutually causal, forming a vicious circle, and ultimately leading to irreversible damage to cardiomyocytes. During the MIRI, pyroptosis is accompanied by inflammation, and ferroptosis is related to apoptosis caused by ERS (Toldo et al., 2018; Li W. et al., 2020). In addition, miRNA can regulate the expression of genes related to oxidative stress, inflammation, energy metabolism disorder, apoptosis, and calcium overload. Therefore, the mechanism of MIRI involves multiple factors and multiple levels, and these factors are interrelated and synergistic, which together lead to serious myocardial tissue damage.

## Saponins in the Treatment of Myocardial Ischemia–Reperfusion Injury

### Protective Mechanism of Saponins From *Ginseng* Against Myocardial Ischemia–Reperfusion Injury

Ginsenosides are essential bioactive ingredients of Araliaceae plants, such as *Panax ginseng* C. A. Mey., *Panax notoginseng* (Burk.) F. H. Chen, and *Panax quinquefolium* L., as well as Cucurbitaceae plants such as *Gynostemma pentaphyllum* (Thunb) Makino (Hu et al., 2010; Shin et al., 2015). *Panax ginseng* C. A. Mey. and *Panax notoginseng* (Burk.) F. H. Chen are the two main plant sources of ginsenosides. Among them, authentic *Panax ginseng* C. A. Mey. is mainly distributed in Southwest China, East Asia and North America, and authentic *Panax notoginseng* (Burk.) F. H. Chen is mainly distributed in Southwest China. Although the proportion of ginsenosides in these Chinese herbal medicines varies, all of them are excellent TCMs for anti-tumor, anti-oxidation, anti-aging, anti-fatigue, regulating blood sugar balance, improving cardiovascular and cerebrovascular, and enhancing immunity (Chen et al., 2011; Wang et al., 2012). Xuesaitong, the main active ingredient of *Panax notoginseng* saponins, is currently used in the clinical treatment of cardiovascular and cerebrovascular diseases and activates blood circulation, alleviates blood stasis, and expands blood vessels (Li et al., 2019). Studies show that Xuesaitong injection can reduce MIRI by promoting pyruvate dehydrogenase-mediated aerobic metabolism (Zhao X. et al., 2017). Ginseng total saponins can also enhance myocardial energy metabolism by regulating the tricarboxylic acid cycle pathway as well as reduce MIRI by inhibiting inflammation and oxidative damage (Wang et al., 2012). *Panax notoginseng* saponins were found to have a protective effect against rat MIRI and cardiomyocyte hypoxia-reoxygenation (HR) by regulating autophagy and apoptosis via the HIF-1α/BNIP3 and PI3K/Akt pathways (Chen et al., 2011) (Table 1).

**TABLE 1:** Anti-MIRI effects of saponins from *ginseng*.

Compound	Major plant source	Geographical distribution of plants	Dose/concentration	Models	Mechanism
Ginseng total saponins	*Panax ginseng* C. A. Mey	Southwest China, East Asia and North America	100, 200 mg/kg, i.g.	Guinea pig MIRI model (*in vivo*) (Aravinthan et al., 2015)	Anti-oxidative and anti-inflammatory properties by reducing inflammatory cytokines and NF-kB
			50 mg/L for 60 min	Rat global MIRI model (*ex vivo*) (Wang et al., 2012)	Modulating TCA cycle protein expression to enhance cardiac energy metabolism; reducing oxidative stress
*Panax notoginseng* saponins	*Panax notoginseng* (Burk.) F. H. Chen	Southwest China	200, 500 μg/ml	Neonatal rat MIRI model (*in vitro*) (Wang et al., 2019)	Inhibiting oxidative stress via MiR-30c-5p
			30, 60 mg/kg, i.p.	Rat MIRI model (*in vivo*) (Liu X.-W. et al., 2019)	Regulating the HIF-1a/BNIP3 pathway of autophagy
			30, 60 mg/kg, i.g.; 0.05, 0.25, 2.25 mg/ml	Rat MIRI model (*in vivo*); H9c2 cardiomyocytes HR model (*in vitro*) (Chen et al., 2011)	Inhibiting apoptosis by activating PI3K/Akt pathway
Gypenoside	*Panax notoginseng* (Burk.) F. H. Chen; *Gynostemma pentaphyllum* (Thunb.) Mak	Southwest China; East Asia and Southeast Asia	50, 100, 200 mg/kg, i.g.; 5, 10, 20 μM	Rat MIRI model (*in vivo*); H9c2 cardiomyocytes HR model (*in vitro*) (Yu et al., 2016a; Yu et al., 2016b)	Inhibiting ER-stress and apoptosis via CHOP pathway and PI3K/Akt pathway; inhibiting NF-kB p65 activation via MAPK signaling pathway
			100 mg/kg, i.g. (*in vivo*); 10, 20 μM (*in vitro*)	Rat MIRI model (*in vivo*); H9c2 cardiomyocytes HR model (*in vitro*) (Chang et al., 2020)	Suppressing miR-143-3p level via the activation of AMPK/Foxo1 signaling pathway
Ginsenoside Rb1	*Panax ginseng* C. A. Mey.; *Panax notoginseng* (Burk.) F. H. Chen	Southwest China, East Asia and North America; Southwest China	40 mg/kg, i.g.	Rat MIRI model (*in vivo*) (Xia et al., 2011; Li et al., 2016)	Enhancing eNOS expression and NO content and inhibiting p38-MAPK signaling pathway
			20, 40, 80 mg/kg, i.g.; 1, 5, 10, 20 μM	Rat global MIRI model (*ex vivo*); rat MIRI model (*in vivo*) (Li C. Y. et al., 2020)	Activating mTOR signal pathway
			2.5, 5, 7.5 mg/kg, i.g.	Rat MIRI model (*in vivo*) (Cui et al., 2017)	Regulating energy metabolism by RhoA signaling pathway
Ginsenoside Rb3	*Panax ginseng* C. A. Mey.; *Panax notoginseng* (Burk.) F. H. Chen	Southwest China, East Asia and North America; Southwest China	2, 5 μM	H9c2 cardiomyocytes HR model (*in vitro*) (Ma et al., 2014)	Inhibiting JNK-mediated NF-kB activation
			5, 10, 20 mg/kg, i.g.	Rat MIRI model (*in vivo*) (Shi et al., 2011; Liu et al., 2013)	Anti-oxidantive, anti-apoptotic and anti-inflammatory activity; improving microcirculatory
Ginsenoside Rd	*Panax ginseng* C. A. Mey.; *Panax notoginseng* (Burk.) F. H. Chen	Southwest China, East Asia and North America; Southwest China	50 mg/kg, i.p.	Rat MIRI model (*in vivo*) (Zeng et al., 2015)	Activating Nrf2/HO-1 signaling pathway
			50 mg/kg, i.p.; 10 μM	Rat MIRI model (*in vivo*); neonatal rat myocardial cells HR model (*in vitro*) (Wang et al., 2013)	Activating Akt/GSK-3β signaling pathway and inhibiting mitochondria-dependent apoptotic pathway
Ginsenoside Re	*Panax ginseng* C. A. Mey.; *Panax notoginseng* (Burk.) F. H. Chen	Southwest China, East Asia and North America; Southwest China	0.3, 1, 3, 10, 20 μM	Guinea-pig cardiomyocyte electrophysiology (*in vivo*) (Bai et al., 2004)	NO-dependent modulation of the delayed rectifier K^+^ current and the L-type Ca^2+^ current
			30, 100 μM	Rat MIRI model (*in vivo*) (Lim et al., 2013)	Ameliorating the electrocardiographic abnormality and inhibiting TNF-α level
Ginsenoside Rg1	*Panax ginseng* C. A. Mey.; *Panax notoginseng* (Burk.) F. H. Chen	Southwest China, East Asia and North America; Southwest China	5 mg/kg/h, 30 min, i.v	Rat MIRI model (*in vivo*) (Li et al., 2018, Yuan et al. 2019)	Inhibiting apoptosis and modulating energy metabolism through binding to RhoA; activating HIF-1 α-ERK signaling pathways
			100 μM	H9c2 cardiomyocytes HR model (*in vitro*) (ZL et al., 2012; Qin et al, 2018)	Inhibiting autophagosomal formation and apoptosis; activating the PI3K/AKT/mTOR pathways
Ginsenoside Rg3	*Panax ginseng* C. A. Mey.; *Panax notoginseng* (Burk.) F. H. Chen	Southwest China, East Asia and North America; Southwest China	5, 20 mg/kg, i.g.	Rat MIRI model (*in vivo*) (Zhang et al., 2016)	Anti-apoptosis and anti-inflammation properties
			60 mg/kg, i.p.; 10 mM	Rat MIRI model (*in vivo*); neonatal rat myocardial cells HR model (*in vitro*) (Wang Y. et al., 2015)	Regulating Akt/eNOS signaling pathway and Bcl-2/Bax signaling pathway
Notoginsenoside R1	*Panax notoginseng* (Burk.) F. H. Chen	Southwest China	5, 10, 20 μM	Rat global MIRI injury model (*ex vivo*); H9c2 cardiomyocytes HR model (*in vitro*) (Yu et al., 2016c)	Inhibiting oxidative stress and ERS
			5 mg/kg, i.g.; 10, 100 nM	Rat MIRI model (*in vivo*); H9c2 cardiomyocytes HR model (*in vitro*) (He et al., 2014)	Preventing energy metabolism disorder via inhibiting ROCK
			20, 40, 60 mg/kg, i.g.	Rat MIRI model (*in vivo*)	Regulating vitamin D3 upregulated protein 1/NF-κB signaling pathway

**FIGURE 2:**
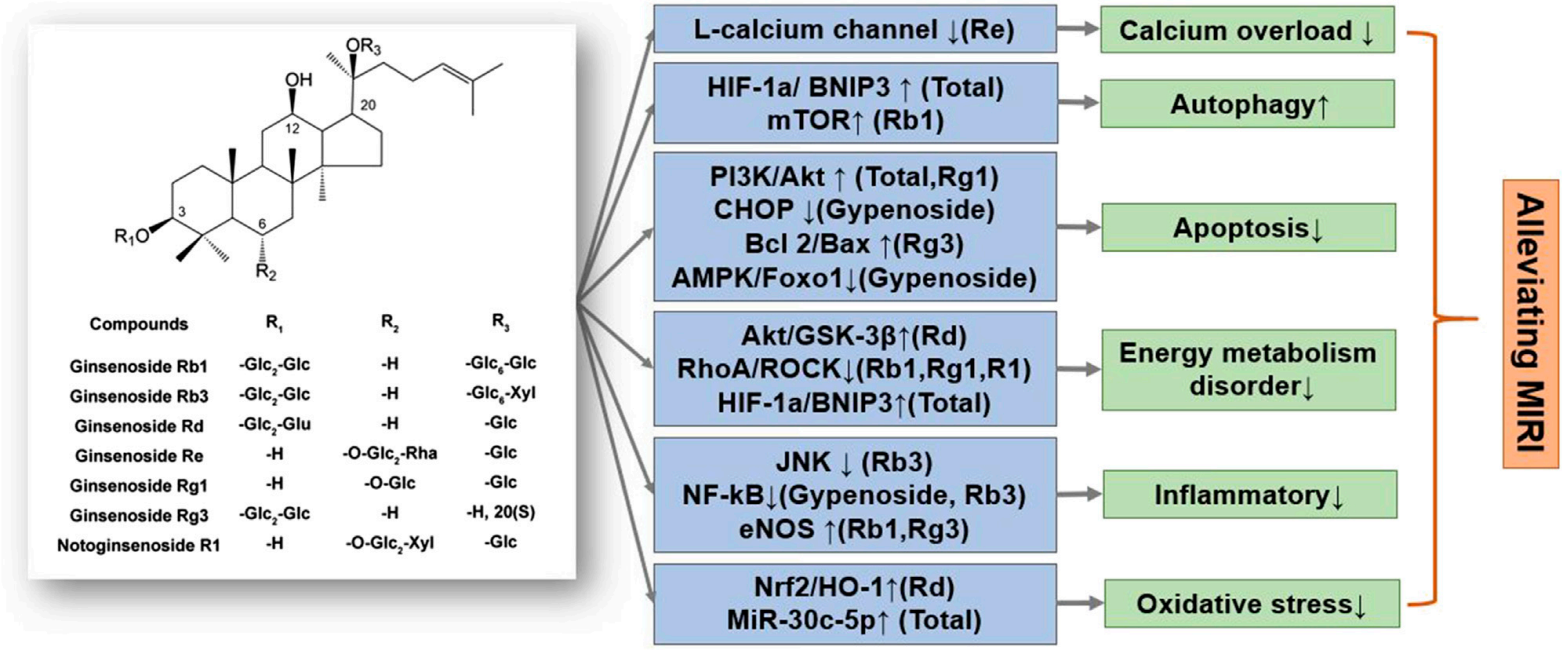
A schematic representation of mechanisms of saponins from *Ginseng* exerted cardioprotective effects in MIRI. Glc, glucose; Rha, rhamnose; Xyl, xylopyranose. ↑means activate relevant pathways. ↓means suppress relevant pathways.

Ginsenosides, such as ginsenoside Rb1, ginsenoside Rg1, ginsenoside Rb3, ginsenoside Rg3, ginsenoside Rd, and ginsenoside Re, are mainly dammarane-type structures, with different types and positions of glycosides that result in unique physicochemical and biological activities (Aravinthan et al., 2015; Liu X.-W. et al., 2019) (Figure 2). Ginsenoside Rb1, a common saponin in *Panax ginseng* C. A. Mey., *Panax notoginseng* (Burk.) F. H. Chen, and *Panax quinquefolium* L., has an excellent therap effect on MIRI *in vivo* and *ex vivo*, mainly by inhibiting apoptosis pathways and activating mTOR phosphorylation (Li C. Y. et al., 2020). Moreover, ginsenoside Rb1 as well as ginsenoside Rg1, improved heart function by improving energy metabolism via the RhoA signaling pathway, which is similar to the cardiac regulation by ginseng total saponins (Cui et al., 2017). Ginsenoside Rg1 also protected H9c2 cardiomyocytes by inhibiting apoptosis, or activating the PI3K/AKT/mTOR pathway (Deng et al., 2015; Li et al., 2018; Qin et al., 2018; Yuan et al., 2019). In addition, ginsenoside Rd restored mitochondrial damage and inhibited oxidative stress by activating the Akt/GSK-3β and Nrf2/HO-1 signaling pathways (Wang et al., 2013; He et al., 2014; Yu et al., 2016c). Notoginsenoside R1 reduced MIRI by preventing energy metabolism disorder and ERS, which was related to the ROCK and NF-κB signaling pathways.

**FIGURE 3:**
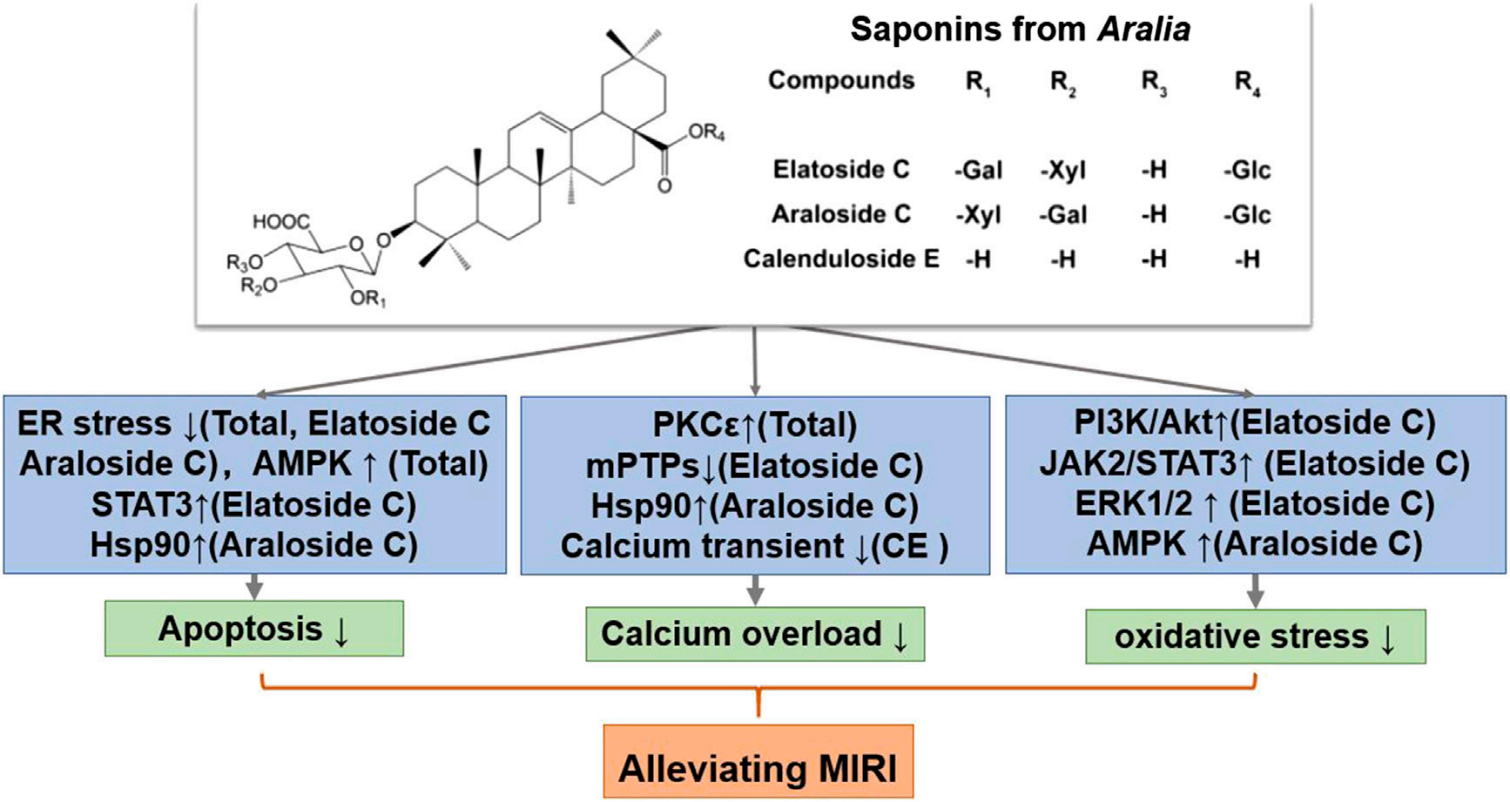
A schematic representation of mechanisms of saponins from *Aralia* exerted cardioprotective effects in MIRI. Glc, glucose; Gal, galactose; Xyl, xylopyranose. ↑means activate relevant pathways. ↓means suppress relevant pathways.

In summary, total ginsenoside as well as ginsenoside monomer components can significantly reduce MIRI and HR damage. The specific regulatory mechanisms of ginsenosides mainly reduce energy metabolism disorders, inhibit oxidative stress and inflammatory response, and reduce cardiomyocyte apoptosis (Li et al., 2016; Zhang et al., 2016). Notably, the regulation of energy metabolism by ginsenosides, mainly through the Akt/GSK-3β and RhoA/ROCK signaling and mitochondrial autophagy pathways, is particularly significant and coincides with the significance of ginseng’s TCM guidance (Figure 3). Various ginsenosides can protect against MIRI through the regulation of different signaling pathways. The clinical efficacy of ginsenosides also encourages researchers to further explore the mechanism underlying the treatment of cardiovascular diseases, provide a theoretical basis for their clinical application, and expand ginseng indications.

### Protective Mechanism of Saponins From *Aralia* Against Myocardial Ischemia–Reperfusion Injury

*Aralia chinensis* L. is an edible Araliaceae plant with medicinal value, including *Aralia elata* (Miq) Seem. and *Aralia taibaiensis* (Wang et al., 2014). *Aralia elata* (Miq) Seem. is mainly distribution in Northeast China, North Korea, Japan and Russia, and *Aralia taibaiensis* is distribution in Midwest China. It contains several triterpene saponins, flavonoids, coumarins, and alkaloids, of which saponins are its main active ingredient (Wang R. et al., 2018). Current research shows that *Aralia* saponins can regulate the cardiovascular system and possess anti-tumor and anti-inflammatory effects (Yan et al., 2015). The total saponin of *Aralia elata* constitute the main component of the Longya Guanxinkang capsule, which functions to nourish qi, promote blood circulation, reduce blood stasis, and relieve pain; it is suitable for treating coronary heart disease, angina pectoris with qi deficiency, and blood stasis syndrome (Wang R. et al., 2018). In addition, *Aralia* Xinmaitong capsules, developed with total saponins of *Aralia elata* as ingredients, have obtained drug clinical research approval for the treatment of angina pectoris caused by qi deficiency and blood stasis (Wang M. et al., 2015). Our team has previously proved that total saponins of *Aralia elata* (Miq) Seem. protects against MIRI by suppressing ERS-related apoptosis and calcium overload via the PKCε-dependent signaling pathway (Wang R. et al., 2018). Total saponins of *A*. *taibaiensis* also showed a protective effect against MIRI (*in vivo*) and HR (*in vitro*), and the protective mechanism was associated with AMPK pathway-related apoptosis (Yan et al., 2015).

**FIGURE 4:**
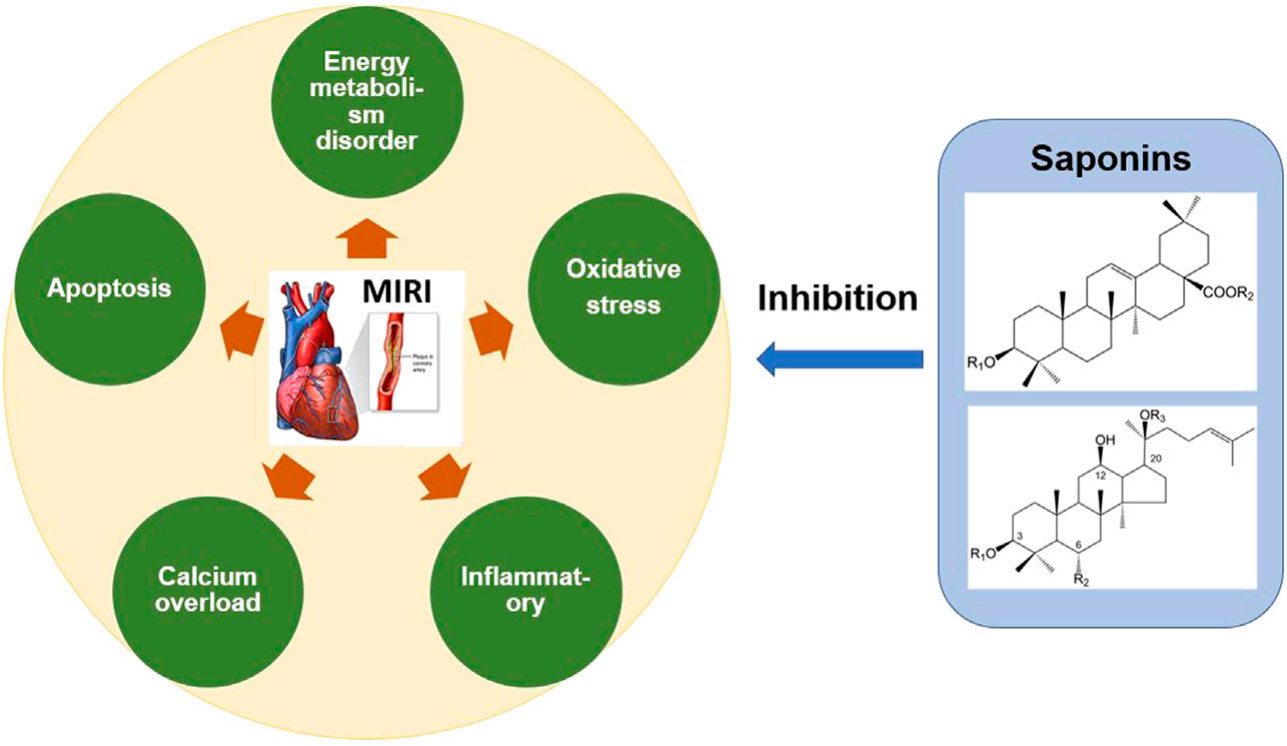
Mechanism of anti-MIRI of saponins in Chinese Herbal Medicine. The saponins in *Panax ginseng*, *Panax notoginseng*, *Aralia*, *Astragalus* and other Chinese herbal medicine can significantly alleviate MIRI. These saponins inhibit oxidative stress, energy metabolism disorder, calcium overload, inflammation and apoptosis, and thus exert the cardioprotective effect.

After screening a large number of saponin components, three components with strong anti-MIRI activity were identified: elatoside C, araloside C, and calenduloside E (Table 2). The chemical structures of these saponins have oleanolic acid configurations (Figure 4). Calenduloside E contains fewer glycosidic bonds than elatoside C and araloside C, which results in its marginal inferior solubility and different biological activities. Further, calenduloside E reportedly reduced H_2_O_2_-induced H9c2 cardiomyocyte injury by inhibiting oxidative stress, apoptosis, and calcium overload (Tian et al., 2017). Elatoside C protected against rat global MIRI by attenuating oxidative stress and calcium overload via PI3K/Akt and JAK2/STAT3 signaling pathway activation and MPTP inhibition (Wang M. et al., 2015). Araloside C reduced oxidative stress, ERS, and calcium overload by regulating Hsp90, and improved mitochondrial function and AMPK activation, which were dedicated to alleviating rat global MIRI and H9c2 cardiomyocyte HR damage (Wang et al., 2017; Du et al., 2018; Wang et al., 2019).

**TABLE 2:** Anti-MIRI effects of saponins from *Aralia*.

Compound	Major plant source	Geographical distribution of plants	Dose/Concentration	Models	Mechanism
Total saponins of *Aralia elata* (Miq) Seem	*Aralia elata* (Miq) Seem	Northeast China, North Korea, Japan, Russia	25, 50, 100 mg/kg, i.g.	Rat MIRI model (*in vivo*) (Wang R. et al., 2018)	Alleviating calcium homeostasis imbalance and endoplasmic reticulum stress-related apoptosis
			30, 60 mg/kg; 1.25, 2.5 and 5 mg/ml	Dog hemodynamic indexes (*in vivo*), Ca^2+^ transients and sarcomere shortening detection (*in vitro*) (Wang et al., 2014)	Positive inotropic effect by maintenance of calcium homeostasis and increase of PKCε-dependent signaling pathway
Total saponins of *Aralia taibaiensis*	*Aralia taibaiensis*	Midwest China	60, 120, 240 mg/kg, i.g.; 25, 50 μg/ml	Rat MIRI model (*in vivo*); H9c2 cardiomyocytes HR model (*in vitro*) (Yan et al., 2015)	Suppressing apoptosis via the AMPK pathway
Elatoside C	*Aralia elata* (Miq) Seem	Northeast China, North Korea, Japan, Russia	2, 10, 50 nM	Rat global MIRI injury model (*ex vivo*) (Wang M. et al., 2015)	Attenuating oxidative stress and calcium overload through the activation PI3K/Akt and ERK1/2 and JAK2/STAT3 signaling pathway and inhibiting the opening of mPTPs
			12.5, 25, 50 μM	H9c2 cardiomyocytes HR model (*in vitro*) (Wang et al., 2014)	Activating STAT3 signaling pathway and reducing ER stress-associated apoptosis
Araloside C	*Aralia elata* (Miq) Seem	Northeast China, North Korea, Japan, Russia	0.5, 1, 2.5 μM; 3.125, 6.25, 12.5, 25 μM	Rat global MIRI injury model (*ex vivo*); H9c2 cardiomyocytes HR model (*in vitro*) (Wang et al., 2017; Du et al., 2018)	Reducing oxidative stress, ER stress and calcium overload by regulating Hsp90
			6.25, 12.5, 25 μM	H_2_O_2_-induced H9c2 cardiomyocyte injury (*in vitro*) (Wang et al., 2019)	Reducing oxidative stress by regulating mitochondrial function and AMPK activation
Calenduloside E	*Aralia elata* (Miq) Seem	Northeast China, North Korea, Japan, Russia	0.02, 0.1, 0.5 μg/ml	H_2_O_2_-induced H9c2 cardiomyocyte injury (*in vitro*) (Tian et al., 2017)	Inhibiting oxidative stress, apoptosis and calcium overload

In short, *Aralia* saponins have an excellent effect on the treatment of coronary heart disease and angina pectoris (Wang R. et al., 2018). Current studies have shown that the cardioprotective effect of *Aralia* saponins is mainly through inhibition of calcium overload, oxidative stress and ER stress-related apoptosis. *Aralia* saponins showed a significant role in maintaining calcium homeostasis, primarily by activating HSP90, PKCε and JAK2/STAT3 signaling pathways, inhibiting MPTP opening (Wang et al., 2014; Wang M. et al., 2015; Du et al., 2018) (Figure 3). However, the current research on MIRI protective mechanism of *Aralia* saponins is not deep enough, and some data on animals *in vivo* experiments and clinical studies are insufficient.

### Protective Mechanism of Other Saponins Against Myocardial Ischemia–Reperfusion Injury

In addition to ginsenosides and aralia saponins, other saponins also possess anti-MIRI activity (Table 3). Since each saponin has a different chemical structure, its characteristic cardiac protection mechanism also differs. *Radix Astragali* is a known TCM for nourishing qi and is widely used in prescriptions and health products. Pharmacological studies have confirmed that *Radix Astragali* has a significant effect on the immune as well as cardiovascular systems (Tu et al., 2013). Huangqi Tongbi decoction, an ancient prescription for treating coronary heart disease that has *Radix astragali* as the main active ingredient, has been shown to significantly improve MIRI in rats; its cardiac protection mechanism involves inflammation suppression via the HMGB1/TLR/NF-κB pathway (Liu K. et al., 2019). In addition, several studies on the anti-MIRI effect of astragaloside IV have identified it as the main component of *Astragalus* responsible for exerting cardiac protection. The mechanism of cardioprotection by astragaloside IV mainly focuses on the improvement of energy metabolism disorders by recovering mitochondrial respiration, preventing MPTP opening, and decreasing cytochrome C release (Xu et al., 2008; Han et al., 2011; Tu et al., 2013). The structure of astragaloside IV is similar to that of dammarane-type ginsenosides, and both possess the same cardiac protection mechanism, which supports the idea of structure determines function.

**TABLE 3:** Anti-MIRI effects of other saponins.

Compound	Major plant source	Geographical distribution of plants	Dose/concentration	Models	Mechanism
Astragaloside IV	*Astragalus membranaceus* (Fisch.) Bge.	Northern China	1, 10 mg/kg, i.g.	Rat MIRI model (*in vivo*) (Tu et al., 2013)	Regulating energy metabolism
			3, 10, 30 μM	Neonatal rat myocardial cells HR model (*in vitro*) (Xu et al., 2008)	Improving intracellular calcium handling via regulating SERCA
			5 mg/kg, i.g.; 10, 20, 40, or 80 μM	Rat global MIRI injury model (*ex vivo*); H9c2 cardiomyocytes HR model (*in vitro*) (Luo et al., 2019)	Recovering mitochondrial respiration, preventing mPTP opening, decreasing cytochrome C release and preventing apoptosis; regulating KATP channel
			0.1, 1, 10, 100 μM	H9c2 cardiomyocytes HR model (*in vitro*) (Yang et al., 2018)	Regulating PI3K/Akt/HO-1 signaling pathway
			80 mg/kg, i.g.; 60 μM	Rat MIRI model (*in vivo*); H9c2 cardiomyocytes HR model (*in vitro*) (Yin et al., 2019)	Inhibiting CaSR/ERK1/2 and the related apoptotic signaling pathways; regulating energy metabolism
Betulinic acid	*Syzygium jambos* (L.) Alston; *Betula platyphylla* Suk.	China, Indochina, Malaysia, Indonesia; China, Russia, Mongolia	50, 100, 200 mg/kg, i.g.	Rat MIRI model (*in vivo*) (Xia et al., 2011)	Preventing cardiomyocyte apoptosis by reducing the release of LDH and CK
			5, 10, 20 μM	H9c2 cardiomyocytes HR model (*in vitro*) (Wang D. et al., 2018)	Inhibiting oxidative stress and apoptosis by Nrf2/HO-1, p38 and JNK pathways
Celastrol	*Tripterygium wilfordii* Hook.F.	East Asia	50 nM	H9c2 cardiomyocytes HR model (*in vitro*) (Li et al., 2017)	Inhibiting the activation of NF-κb
			0.01, 0.1, 1 μM	H9c2 cardiomyocytes HR model (*in vivo*); rat global MIRI injury model (*ex vivo*) (Aceros et al., 2019)	Modulating HSP90 activity
			2, 4, 6 mg/kg, i.g.	Rat MIRI model (*in vivo*) (Tong et al., 2018)	Suppressing apoptosis, inflammatory and oxidative stress via PI3K/Akt pathway activation and HMGB1 inhibition
Clematichinenoside	*Clematis chinensis* Osbeck	China, Vietnam	1, 10, 100 μM	H9c2 cardiomyocytes HR (*in vitro*) (Ding et al., 2016)	Inhibiting apoptosis through mitochondrial mediated apoptotic signaling pathway
			0.001, 0.01, 0.1 mg/ml; 8, 16, 32 mg/kg, i.g.; 1, 10, 100 μM	Rat global rat MIRI injury model (*ex vivo*); rat MIRI model (*in vivo*); neonatal rat myocardial cells HR (*in vitro*) (Zhang et al., 2013)	Restoring an antioxidant effect by restoring the balance between inducible NO synthase and endothelial NO synthase
Dioscin	*Dioscorea oppositifolia* L	China, Japan, South Korea	0.1, 1 nM	Rat global MIRI injury model (*ex vivo*) (Badalzadeh et al., 2014; Badalzadeh et al., 2015)	Activating mitochondrial K_ATP_ channels and NO system, attenuating oxidative stress
			50, 100 mg/kg, i.g.	Rat MIRI model (*in vivo*) (Wang H. W. et al., 2018)	Inhibiting inflammation by regulating p38-MAPK/JNK pathways and NF-κb pathways
Glycyrrhizin	*Glycyrrhiza uralensis* Fisch.	China, Russia	2, 4, 10 mg/kg, i.g.	Rat MIRI model (*in vivo*) (Han et al, 2011; Zhai et al., 2012)	Inhibiting oxidative stress, iNOS and inflammatory, via HMGB1 and MAPK expression
			5, 10, 20 mg/kg, i.g.	Rat MIRI model (*in vivo*) (Wu et al., 2015)	Prolonging APD, inhibiting I_ca-L_ and I_to_; blocking phospho-JNK/Bax pathway
Ilexsaponin A	*Ilex pubescens* Hook. et Arn.	China	10, 40 mg/kg, i.g.; 10, 50, 250 μM	Rat MIRI model (*in vivo*); neonatal rat myocardial cells HR model (*in vitro*) (Zhang et al., 2017)	Inhibiting apoptotic pathway
Ophiopogonin D	*Ophiopogon japonicus* (Linn. f.) Ker-Gawl.	China, Japan, Vietnam, India	20 mg/kg, i.p.	Rat MIRI model (*in vivo*) (Huang et al., 2018)	Antioxidant and anti-apoptotic effects
Sasanquasaponin	*Camellia oleifera* Abel	Southern China	0.1, 1, 10 μM	Neonatal rat myocardial cells HR model (*in vitro*) (Chen et al., 2007)	Inhibiting oxidative stress via attenuating ROS generation and increasing antioxidant activities
			0.1, 0.2, 0.4 mg/kg, i.g.; 0.1 μM	Mouse MIRI model (*in vivo*); adult mouse myocardial cell HR model (*in vitro*) (Lai et al., 2004)	Modulating intracellular Cl- homeostasis and anti-arrhythmia effects
Withaferin A	*Withania Somnifera*	India	1, 5 mg/kg, i.g.	Rat MIRI model (*in vivo*) (R. et al., 2019)	Upregulating AMP-activated protein kinase-dependent B-cell lymphoma2 signaling
			0.1, 1 μM	Neonatal rat myocardial cells HR model (*in vitro*) (Yan et al., 2018)	Inhibiting apoptosis via activated Akt-mediated oxidative stress suppression

Dioscin is a phytoestrogen that exhibits anti-MIRI activity. Diosgenin exerts cardioprotective effects by inhibiting inflammation and oxidative stress by activating mitochondrial K_ATP_ channels and regulating p38-MAPK/JNK pathways (Badalzadeh et al., 2014; Badalzadeh et al., 2015; Wang H. W. et al., 2018). Estrogen and its receptors are both critical targets in the MIRI mechanism; therefore, dioscin can also be used as a drug candidate for the treatment of MIRI, and its cardioprotective mechanism needs further research. Celastrol, another anti-MIRI saponin, can interact with HSP90, which is similar to *Aralia* saponins. The structure of celastrol has more unsaturated bonds than oleanolic acid, which indicates its superior antioxidant activity (Aceros et al., 2019). Previous studies have also confirmed that cardiac heart protection mechanism of celastrol involves suppression of oxidative stress, inflammation, and apoptosis via the PI3K/Akt and HMGB1 pathways (Li et al., 2017; Tong et al., 2018). Some studies have suggested that high-mobility group box 1 (HMGB1) plays a role in early MIRI, activates the inflammatory response, and promotes cardiomyocyte apoptosis (Tong et al., 2018). Glycyrrhizin, the main active compound in licorice, is a natural HMGB1 inhibitor. Studies have also shown that glycyrrhizin reduces rat MIRI by inhibiting oxidative stress and inflammation (Zhai et al., 2012; Wu et al., 2015).

Furthermore, anti-MIRI saponins also include betulinic acid, clematichinenoside, ilexsaponin A, ophiopogonin D, sasanquasaponin, and withaferin A. Their cardiac protection mechanisms are different, and mainly involve suppression of oxidative stress, inflammation, and apoptosis (Huang et al., 2018; Yan et al., 2018). Based on the current research progress, it can be concluded that the signaling pathways involved in the regulatory mechanism of each saponin are different and that one saponin may affect one or more signaling pathways, indicating the diversity of their therapeutic targets. Saponins with different structures regulate various signaling pathways to achieve cardioprotection. However, the current research on the anti-MIRI effect of saponins is not comprehensive, requiring researchers to continue their exploration so as to provide a more theoretical basis and reliable drug candidates for MIRI treatment.

## Conclusions and Perspectives

The targets and anti-MIRI mechanisms of saponins are diverse and mainly include inhibition of oxidative stress, calcium overload, inflammation, and mitochondrial dysfunction. In brief, saponins have a strong antioxidant effect, which in turn helps restore mitochondrial function and intracellular calcium homeostasis, reduces the production of inflammatory factors, and inhibits cardiomyocyte apoptosis, thereby exerting a cardioprotective function (Zhang et al., 2016; Zhang et al., 2017; Yin et al., 2019). Recent studies have revealed the important roles of pyroptosis and ferroptosis in the pathogenesis of MIRI (Wang Z. et al., 2018; Li W. et al., 2020). However, there is no relevant research on the effects of saponins on pyroptosis and ferroptosis. Therefore, the regulatory role of saponins on these new MIRI mechanisms needs to be further studied to completely elucidate the protective mechanism of saponins against MIRI.

Structurally, saponins with vigorous biological activity are mainly oleanolic acid saponins and dammarane-type saponins. Studies have shown that oleanolic acid saponins have a significant regulatory effect on calcium homeostasis (Wang M. et al., 2015), whereas dammarane-type saponins have a more substantial regulatory effect on energy metabolism (Cui et al., 2017). In addition, ginsenosides are more active in dammarane-type saponins, while *Aralia* saponins are more active in oleanolic acid saponins, which further proves that the unique characteristics of TCM are determined by the structure of its key active components. The occurrence of MIRI is a multi-factor interaction and an extremely complicated process; therefore, multi-target therapy will be the future direction for drug development. Saponins in TCM can act on multiple pathways simultaneously and effectively reduce MIRI (Figure 4). Thus, saponins provide a broad application prospect for the development of highly effective and low-toxicity anti-MIRI drugs.

At present, the study of saponins is still in its initial stage of new structural saponin discovery and data accumulation. Research on the structure–activity relationship of saponins against MIRI, at home and abroad, is still in its infancy. Although the different biological activities and mechanisms of saponins have been gradually elucidated at the molecular level, their clinical applications and saponin-treatment studies for MIRI are limited. Therefore, the systematic summary of the anti-MIRI mechanism of saponins can potentially lay a foundation for detailed study of the anti-MIRI effect and structure–activity relationship of saponins, and thereby aid the development of new anti-MIRI drugs with new mechanisms or targets. A detailed study of the structure–activity relationship of saponins against MIRI would enable the identification of active components or monomers of TCM saponins. This would finally aid the development of drugs with more active and less adverse reactions through chemical modification and artificial synthesis. Thus, the study of saponins will become an important research direction in the development of anti-MIRI drugs.

## Author Contributions

Conceptualization, RW, JZ, and MW; writing—original draft preparation, RW; writing—review and editing, MW, GS, and XS; visualization, DW, JY, and JZ; funding acquisition, GS and XS. All authors have read and agreed to the published version of the manuscript.

## Funding

This research was funded by Central Public-Interest Scientific Institution Basal Research Fund (No. 2018PT35030 by GS), the National Natural Science Foundation of China (No. 81973514 by GS), the Drug Innovation Major Project (No. 2018ZX09711001-009 by XS) and National Natural Science Foundation of China (No. 81891012 by XS).

## Conflict of Interest

The authors declare that the research was conducted in the absence of any commercial or financial relationships that could be construed as a potential conflict of interest.
